# EEG Assessment in a 2-Year-Old Child with Prolonged Disorders of Consciousness: 3 Years' Follow-up

**DOI:** 10.1155/2020/8826238

**Published:** 2020-11-21

**Authors:** Gang Xu, Qianqian Sheng, Qinggang Xin, Yanxin Song, Gaoyan Zhang, Lin Yuan, Peng Zhao, Jun Liang

**Affiliations:** ^1^Rehabilitation Branch, Tianjin Children' Hospital, Tianjin 300400, China; ^2^Tianjin Tianshi University Medical College, Tianjin 301700, China; ^3^School of Artificial Intelligence, College of Intelligence and Computing, Tianjin University, Tianjin 300354, China; ^4^Departmemt of Rehabilitation, Tianjin Medical University General Hospital, Tianjin 300041, China; ^5^Lab of Neural Engineering & Rehabilitation, Department of Biomedical Engineering, College of Precision Instruments and Optoelectronics Engineering, Tianjin University, Tianjin 300354, China

## Abstract

A 2-year-old girl, diagnosed with traumatic brain injury and epilepsy following car trauma, was followed up for 3 years (a total of 15 recordings taken at 0, 2, 3, 4, 5, 6, 7, 9, 10, 11, 12, 14, 19, 26, and 35 months). There is still no clear guidance on the diagnosis, treatment, and prognosis of children with disorders of consciousness. At each appointment, recordings included the child's height, weight, pediatric Glasgow Coma Scale (pGCS), Coma Recovery Scale-Revised (CRS-R), Gesell Developmental Schedule, computed tomography or magnetic resonance imaging, electroencephalogram, frequency of seizures, oral antiepileptic drugs, stimulation with subject's own name (SON), and median nerve electrical stimulation (MNS). Growth and development were deemed appropriate for the age of the child. The pGCS and Gesell Developmental Schedule provided a comprehensive assessment of consciousness and mental development; the weighted Phase Lag Index (wPLI ) in the *β*-band (13–25 Hz) can distinguish unresponsive wakefulness syndrome from minimally conscious state and confirm that the SON and MNS were effective. The continuous increase of delta-band power indicates a poor prognosis. Interictal epileptiform discharges (IEDs) have a cumulative effect and seizures seriously affect the prognosis.

## 1. Introduction

The development of emergency and critical care medicine is changing rapidly. After severe brain damage caused by trauma, patients may experience coma, unresponsive wakefulness syndrome (UWS), minimally conscious state (MCS) [1, 2], and long-term motor and cognitive impairment. The prevalence of UWS is between 0.2 and 3.4 per 100,000 [3, 4]. 10 to 15% of patients (including adults and children) who survived the acute coma stage entered a state of prolonged disorders of consciousness (PDOC) [5, 6]. The incidence in children is particularly prominent due to a lack of self-protection awareness and the errors of parents or caregivers [7]. In the U.S., between 4,000 and 10,000 children each year suffer from a persistent vegetative state (now termed UWS). 24% of children in a persistent vegetative state after traumatic brain injury (TBI) regained consciousness after 3 months, while after 12 months, this proportion rose to 62% [8, 9]. 63% of UWS patients survived for more than 8 years [10]. This undoubtedly reduces the patient's quality of life and increases the burden on the family and society.

At present, the diagnosis and treatment of disorders of consciousness caused by severe brain injury are still very difficult, especially in children [11]. Further, there is no clear guidance regarding diagnosis, treatment, or prognosis. Multisensory stimulation is widely recommended because of an extremely low adverse reaction rate and the presence of observable immediate reactions and because it facilitates active family participation. However, the technique lacks any evidence of efficacy [12, 13]. Electroencephalogram (EEG) is the only electrophysiological recording technique available in most primary hospitals. EEG can be used to provide periodic and repeated evaluations at the bedside to track the patient's rehabilitation trajectory [14]. EEG analysis can give information related to a child's consciousness level and epilepsy activity and complements brain imaging research. EEG analysis has been widely used in research in the field of disorders of consciousness [15–19]. In this paper, EEG analysis is used to longitudinally observe the diagnosis and treatment process in a severe brain injury case, which may provide a reference for clinical research.

## 2. Materials and Methods

### 2.1. Case

A 2-year-old girl presented having suffered from prolonged disorders of consciousness (PDOC) for 2 months and was diagnosed with traumatic brain injury and epilepsy. The girl's development before the injury was normal.

On attending the emergency department after the car accident, a pediatric Glasgow Coma Scale (pGCS) of 3 was recorded. CT (computed tomography) showed a high-density shadow in the right temporal lobe representing a small amount of cerebral parenchymal hematoma. Also observed were subarachnoid hemorrhage and bilateral traumatic wet lung, but no abnormalities in the abdominal pelvis. Neurosurgery provided ventilator-assisted breathing, a blood transfusion was given to increase blood volume, in addition to mannitol and dexamethasone, and other nonsurgical protective treatments to reduce cerebral edema. After the vital signs had become stable, the patient was transferred to the rehabilitation department to continue treatment. At this point, a pGCS of 8 was recorded (E4-V2-M2) in addition to quadriplegia.

Head magnetic resonance imaging (MRI) showed bilateral frontotemporal apical subdural effusion, white matter areas with high signal intensity in the bilateral parietal lobe, low signal shadows in the bilateral cerebellar hemispheres, brainstem, and cistern of the foot signified contusion and laceration and possible subarachnoid hemorrhage, and long *T*_1_ and long *T*_2_ signals in the right lateral basal ganglia area signified hemorrhagic absorption, and there was a notable widening of the ventricle and extraventricular space.

**Figure 1:**
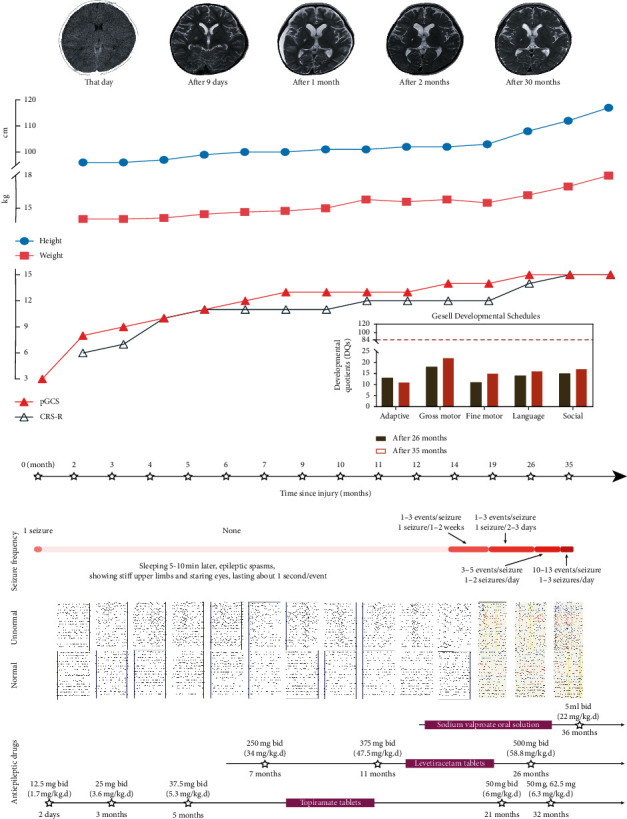
14 consecutive assessments after the onset: pGCS, CRS-R, Gesell, height, weight, seizure frequency, oral antiepileptic medications, CT, and MRI.

The treatment regime comprised (1) maintaining stable vital signs and nutritional support to prevent complications; (2) the administration of various benign stimuli (namely, subject's own name, SON; electrical median nerve stimulation, MNS; and proprioceptive stimulation) and physiotherapy; (3) hyperbaric oxygen cabin treatment; and (4) oral antiepileptic medication as shown in Figure 1.

### 2.2. Assessments

**Figure 2:**
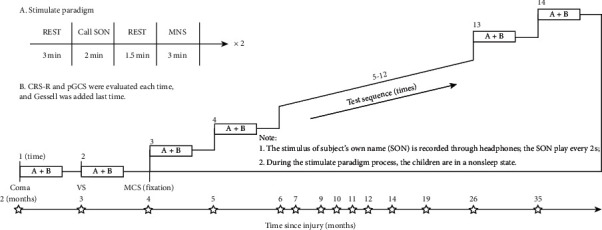
Experimental design.

pGCS, CRS-R, Gesell Developmental Schedule, height, weight, seizure frequency, oral antiepileptic medication, CT, and MRI are shown in Figure 1. The same researchers (comprising 3 rehabilitation physicians and 1 psychological counselor) performed EEG acquisition and observations via video recordings taken in the EEG room or at the bedside. The processed EEG recordings are shown in Figure 2. CRS-R and pGCS were evaluated by three rehabilitation physicians at different times within the same day. The results were based on the same score being given by more than two physicians. Clinical examination, pGCS, and CRS-R assessment (accounting for fluctuations in the functional status of the child) began immediately before the EEG recording. The Gesell test was evaluated by a psychological counselor.

### 2.3. Resting-State EEG

**Figure 3:**
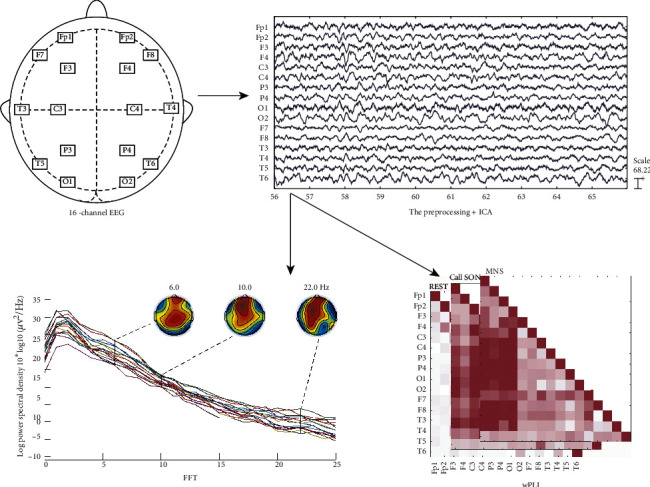
Data processing: after preprocessing and independent component analysis (ICA), the selected EEG epochs were analyzed using fast Fourier transform (FFT) and weighted Phase Lag Index (wPLI).

Resting-state EEG was recorded using 16-channel silver chloride single disc electrodes, placed according to the international 10/20 system. The placement of each channel on the head is shown in Figure 3. Data were collected at a sampling rate of 500 Hz or 512 Hz and were later downsampled to 128 Hz offline. The patient's behaviors and EEG data were monitored online to ensure recordings were free from seizure activity. The test paradigm is shown in Figure 2. SON stimulation was recorded by the child's mother, with 2 seconds between adjacent stimulus repeats, edited by Format Factory, and presented using the EDIFIER K800 headset. MNS was administered using a Shanghai NuoCheng stimulator. A skin electrode (2.5 cm × 2.5 cm) was applied to the patient's right forearm 2 cm above the wrist striate. MNS was administered using direct current in the form of a 300 ms-wide asymmetric square wave, with 13 mA stimulation intensity (with reference to thumb or finger jitter), and 40 Hz frequency. The test was repeated twice [20].

Data were filtered between 1 and 25 Hz and then epoched to 2-second epochs. Epochs containing excessive eye movement or muscular artifacts were rejected using a quasiautomated procedure whereby abnormally noisy channels and epochs were identified by calculating their normalized variance. Further noisy channels and epochs were manually rejected or retained by visual inspection. Data with continuous epilepsy-like activity were excluded (e.g., periodic interictal epileptiform discharges, IEDs). Data were rereferenced to compute the average across all channels. Then use independent component analysis (ICA), which is implemented by the RUNICA tool within EEGLAB [21]. Once relatively artifact-free EEG data had been obtained, functions in MATLAB (version 7.6) were used to conduct the power spectrum and functional connection analyses.

EEG data covering the known frequency bands, *δ*1 (1-2 Hz), *δ*2 (3-4 Hz), *θ*1 (5-6 Hz), *θ*2 (7-8 Hz), *α*1 (9-10 Hz), *α*2 (11-12 Hz), and *β* (13–25 Hz), were analyzed. The selected EEG epochs were processed using a fast Fourier transform (FFT). The weighted Phase Lag Index (wPLI) was used to measure connectivity between each pair of electrodes. wPLI minimizes the effects of volume conduction on the estimation of brain connectivity. The wPLI values across all channel pairs were used to construct symmetric 16 × 16 wPLI connectivity matrices for the *β*-band, as shown in Figure 3.

## 3. Results and Discussion

There is limited literature on the diagnosis and treatment of disorders of consciousness in children [11, 22]. The research methods are mostly cross-sectional. Due to the instability of the consciousness state of patients with prolonged disorders of consciousness, the repeatability (especially during UWS and MCS) is poor. The process of recovery comprises two components: development and plasticity. Longitudinal research is of great significance.

In terms of the child's growth and development, her height and weight were seen to develop at the correct rate. With regards to behavior evaluation (Figure 1), her pGCS gradually increased as disease recovery progressed, until it reached 15 points (the maximum score) after 19 months of onset. Her CRS-R appeared to plateau three times during disease recovery (at 5–9 months, 10–14 months, and 26–35 months). The Gesell assessment showed that all 5 areas were extremely underdeveloped at 26 and 35 months. The mother of the child believes that the child has continued to progress. At 19 months after injury, she has been able to understand and execute simple instructions (such as “sit,” “do not shout,” and “wave goodbye”). Further, the frequency of execution has increased slowly.

**Figure 4:**
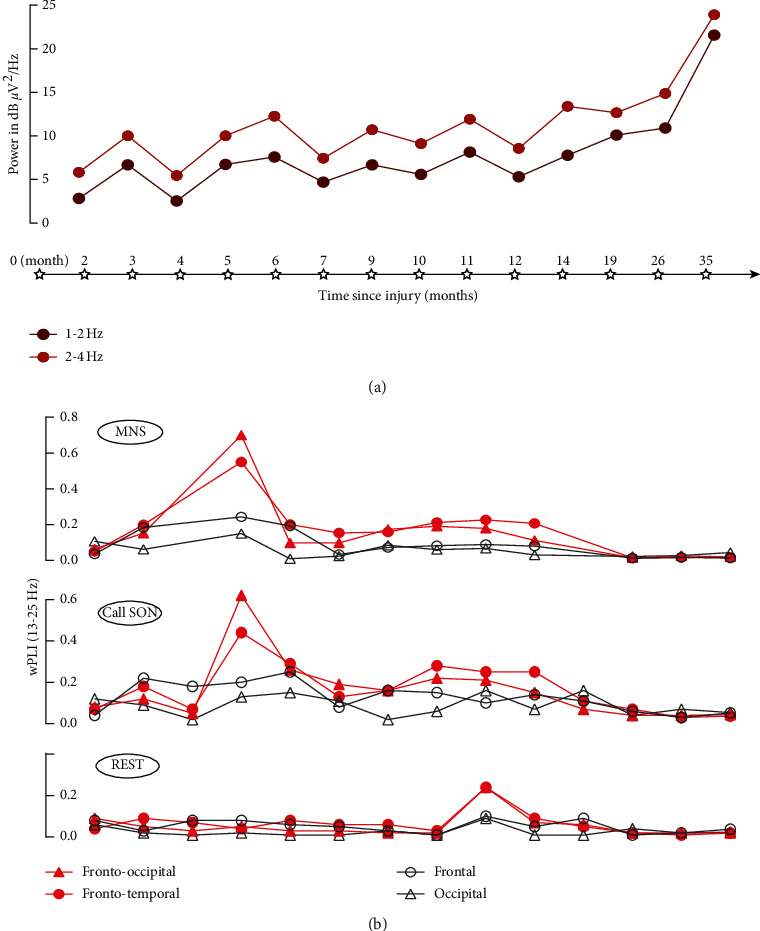
14 consecutive EEG assessments following the injury: (a) FFT and (b) wPLI.

EEG spectral analysis showed the *δ*-band power spectrum continued to increase over the 3 years (Figure 4). At the end of the 3 years, the Gesell assessment showed that all 5 areas were still extremely underdeveloped. This indicated that the continued rapid increase of slow waves may be related to a poor prognosis. It is suggested that the continuous increase of slow waves may be a manifestation of brain dysfunction. The Gesell assessment is more generally used to monitor late neurodevelopment in preterm infants [23], bilirubin encephalopathy [24], and hypoglycemic encephalopathy [25]. Therefore, this study attempts to use the Gesell assessment to monitor the level of neurodevelopment during later follow-up of a child with traumatic brain injury. In follow-up studies related to the prognosis of adult disorders of consciousness, the increase in slow waves has been associated with a poor prognosis [26–29], in agreement with our findings. The decrease of *α* + *β*/*δ* + *θ* is also a common indicator of poor prognosis [18]; however, it was not found during continuous follow-up in this case. One reason for this may be that a case study, being a study of a single individual, is biased. Alternatively, during the rapid development of a 2-year-old child, each frequency band has been shown to change significantly with age [30, 31].

Stimulation using SON and MNS had a positive role in the functional connectivity in the *β* (13–25 Hz) frequency band, as shown in Figure 4. In the UWS period, SON and MNS increased the functional connectivity to a level above that of the resting state. However, there was no obvious difference between long-range functional connectivity and short-range functional connectivity. Counterintuitively, short-range functional connectivity sometimes even exceeded long-range functional connectivity. Over the same period, pGCS and CRS-R scores increased. Therefore, on the one hand, stimulation using SON and MNS may increase cortical functional connectivity in children and improve the state of consciousness, but conversely, short-range functional connectivity still accounts for a large proportion of stimulus responses. This suggests that SON and MNS effectively activate the brain, but that activation is largely concentrated in local brain regions. From the diagnosis of MCS to 19 months after the onset, SON and MNS induced increases in both long-range and short-range functional connectivity to a level above that of the resting state, while pGCS and CRS-R scores also increased. This finding suggests that SON and MNS can significantly increase cortical functional connectivity in children in an MCS and improve the state of consciousness. Between 19 and 35 months following onset, functional connectivity in response to SON and MNS suddenly decreased. This is in agreement with a previous study that demonstrated no significant difference in the short- and long-range functional connectivity in response to stimulation in UWS and MCS. Observations have shown that multisensory stimulation therapy had a more positive effect in unconscious children than in a control group. The early manifestations are reduced agitation and decreased muscle tone, while longer-term manifestations are improved cognitive and self-care abilities [32]. However, no evidence has been provided on the mechanism of action. This study suggests that SON and MNS can increase the short- and long-range cortical functional connectivity in children and provide a partial basis for the mechanism behind the effectiveness of multisensory stimulation.

Epilepsy (including IEDs) was found to play a negative role. IEDs persisted and increased in the first 13 months after injury; however, there were no clinical seizures. Cognitive function significantly improved over this period. Between 13 and 35 months after the injury, spastic seizures appeared and gradually increased while cognitive function stagnated, as shown in Figure 1. Although the dosage and types of antiepileptic drugs had been increased since onset, they did not effectively control the progress of epilepsy. Taking this evidence in combination with the change in *β*-band functional connectivity, it suggests that IEDs have little effect on functional connectivity. However, there may be a cumulative effect that has gone undetected. Seizures had a greater impact on functional connectivity, suggesting that abnormal discharge may be detrimental to brain function. Sometimes, antiepileptic drugs have no obvious effect. The effect of epilepsy on the prognosis of children with disorders of consciousness has not attracted enough attention [22], potentially due to the relatively low incidence of epilepsy in the early stages of these disorders. The incidence of epilepsy within the 5 days following brain trauma is 7%, of which 6% go on to an epilepsy diagnosis [33]. However, after 59 months of follow-up, the incidence of epilepsy reached 20% [34]. It is suggested that children with IEDs or normal EEG in the early stages of brain injury may be likely to develop epilepsy in the long term. The cognitive impairment associated with IEDs is transient or slight, but the cognitive impairment of persistent IEDs can be cumulative [35]. Some studies have shown that the presence of IEDs is an independent risk factor for cognitive impairment in children and may even affect cognitive development through pathological damage to the brain [36, 37]. Controlling IEDs has also been shown to improve cognition and behavior [38]. This serves as a reminder that it is important to pay attention to the management of abnormal discharge. For children with a severe head injury and prolonged increased abnormal discharge, although early identification and diagnosis continue to improve, there is still a possibility of a poor long-term prognosis.

In the entire life cycle of human development, from infants to young children, adolescents, adults, and old age, there are different patterns underlying changes in cognitive and motor abilities [39]. These patterns are affected by the plasticity of the central nervous system. For example, a study found that correct rejection rates (CRRs) improved, and reaction times (RTs) became longer (got worse). Performance increased between a cohort of children to a cohort of young adults but decreased again in the older adult cohort [40]. Observing 586 healthy subjects aged between 2 and 73 years old, it was found that plasticity of the auditory brainstem persisted, especially between 5 and 8 years old [41]. The excitability and plasticity of cells in the cerebral cortex, neural pathways, and neural networks are the key to our exploration of age-related changes in brain structure and function [42]. As a method of electrophysiological monitoring, EEG is one of the most important methods in cognitive neuroscience research [43, 44]. Children with severe neurological diseases may not be able to communicate or interact with their surroundings. Brain-computer interfaces (BCI) provide new opportunities for such children to participate in interactions to improve their quality of life. One study showed that children can quickly achieve control and execute multiple tasks using simple EEG-based BCI systems [45]. This has been demonstrated in autism [46] and cerebral palsy [47]. Therefore, the longitudinal study of pediatric EEG provides a reference for the development of BCI for children. At the same time, it also has important guiding significance for the study of BCI technology for the elderly.

## 4. Limitations

This is a case report. It is challenging to design and conduct a controlled study in this area. The scope of the behavior scale used (the pediatric GCS) is restricted to young children. The Rappaport Coma/Near Coma Scale (CNCS) and Level of Cognitive Functioning Assessment Scale (LOCFAS) [48] may be considered in future studies; however, both these scales are currently used less often and it will take time for clinicians to become familiar with their use. Multimodality assessments, combined with brain imaging, may provide a more advantageous measure.

## 5. Conclusions

In the follow-up of this case, it was found that the continuous increase in the *δ*-band power spectrum indicated a poor prognosis. wPLI in the *β*-band was used to identify the effect stimulation using SON and MNS on cortical functional connectivity. Spectral analyses and wPLI can be used as auxiliary tools for the diagnosis and follow-up of prolonged disorders of consciousness. Interepileptic seizures seriously affect long-term prognosis and need to be actively managed. Long-term continuous follow-up of children with prolonged disorders of consciousness is of great significance in understanding the occurrence and development of these diseases.

## Data Availability

Part of the data used to support the findings of this study may be released upon application to the first author (Gang Xu), who can be contacted via e-mail, xugangrehab@163.com.
